# Epidemiology of intrapartum stillbirth and associated factors among women who gave childbirth in Ethiopia: systematic review and meta-analysis

**DOI:** 10.3389/fgwh.2024.1432729

**Published:** 2024-09-10

**Authors:** Eskinder Israel, Awoke Abraham, Mihiret Tesfaw, Temesgen Geta, Melkamu Worku Kercho, Samson Dubale, Tagese Yakob, Endale Jambo, Eshetu Elfios

**Affiliations:** ^1^ School of Public Health, College of Health Science and Medicine, Wolaita Sodo University, Sodo, Ethiopia; ^2^ Department of Public Health, Marie-stopes International (MSI) Ethiopia Reproductive Choices, Hawassa, Ethiopia; ^3^ School of Nursing, College of Health Science and Medicine, Wolaita Sodo University, Sodo, Ethiopia; ^4^ School of Midwifery, College of Health Science and Medicine, Wolaita Sodo University, Sodo, Ethiopia

**Keywords:** intrapartum, stillbirth, women, childbirth, Ethiopia, systematic review, metaanalysis

## Abstract

**Background:**

Stillbirth always resulted in a multi-dimensional impact from the individual level to the country level at large. It causes psychological depression, social stigmatization, and decreased quality of life for women. Despite several studies conducted in Ethiopia, no national pooled estimates were done. Therefore, this systematic review and meta-analysis sought to assess intrapartum stillbirth and associated factors among women who had childbirth in Ethiopia using the available published evidence.

**Methods:**

The current review included studies conducted in Ethiopia. The databases used primarily were Medline/PubMed, Google Scholar, Scopus, Web of Science, Ethiopian University Repository Online, CINAHL, African Journals Online and Cochrane Library. All cross-sectional studies conducted in English and met eligibility criteria were included in the final review. A random-effects meta-analysis was performed. Data extraction and analysis were also performed using Microsoft Excel and STATA version 14 software respectively.

**Results:**

In the current review, eleven studies were included, and their quality was assessed before being chosen for the final review. The pooled prevalence of intrapartum stillbirth among women who had childbirth in Ethiopia was 9.21% [95% CI (7.03%, 11.39%); *I*^2^ = 90.2, *P* = 0.000]. Women with a previous history of stillbirth [OR = 5.14, 95% CI (3.53–6.75), *I*^2^ = 60.0%, *p *= 0.04] and had no use of antenatal care {[OR = 0.43, 95% CI (0.18–0.68) *I*^2^ = 85.3%, *p* = 0.001]} were significantly associated with intrapartum stillbirth among women who gave childbirth.

**Conclusions:**

Nearly one-tenth of women who had childbirth in Ethiopia had an intrapartum SB. Revitalizing the existing health extension package particularly family health services with emphasis on focused antenatal care and counselling as well as with prompt referral system would reduce intrapartum SB. This review calls for the need to assess the quality of ANC provision and tailor targeted interventions to best improve the service quality.

## Background

World Health Organization (WHO) defines intrapartum stillbirth (SB) as the delivery of a fetus after 28 completed weeks of gestation, or with a birth weight of more than 1,000 gm (01 kg), who had detectable fetal heart sounds during admission, but died during the delivery (1). It is one of the unfavorable outcomes of pregnancy is prevalent in many countries both developed and developing countries despite the presence of good efforts to improve intrapartum care for pregnant women (2, 3). Globally, nearly 2.6 million SBs occurred in 2020, most of which were from South Asia and Sub-Saharan African countries (SSA) (1, 4). These reports further showed that this rate was ten times increased in low resource settings including SSA (26/1,000 LB) when compared with developed countries (6/1000 LB) (5, 6). Recent data also showed that an average of 900,000 pregnancies became SBs every year in 2020 in SSA (7). SSA has the largest share of this figure and has increased from 26% in 2000 to more than 45% in 2021 with a large concentration in Nigeria, the Democratic Republic of Congo and Ethiopia (8, 9).

Results in a multi-dimensional impact from the individual to the country level at large. The growing evidence in the field highlighted that women who suffer stillbirth mostly experience feelings of grief, anxiety, psychological depression, social stigmatization, guilt, pain, post- traumatic stress disorder (PTSD) and decreased quality of life for women (10, 11). The economic costs of intrapartum SBs are largely unknown and often overlooked when compared with other adverse pregnancy outcomes. However, its costs vary from US $4,781–$10,571 per cases averted (in 2013 prices) in low-income and middle-income countries. Despite this, it acts as a sensitive indicator and good reflector of quality obstetric care in pregnancy and childbirth and remains a sensitive marker of the overall health system (11, 12).

Various studies conducted in different settings indicated that poor obstetrical service quality, presence of fetomaternal infections and pregnancy complications including antepartum hemorrhage (APH), pregnancy-induced hypertension (PIH), abusive care during childbirth, Intrauterine fetal growth restriction (IUGR) remained as the most important determinants of intrapartum SB (6, 13–24).

As compared with other countries, Ethiopia has the highest number of SB since 2020 (2, 3, 25). Trends in the Ethiopian National Demographic and Health Survey (DHS) report showed that intrapartum SB rates of 10, 17 and 11 per 1,000 births were reported in 2000, 2011 and 2016 respectively (26).

The government has no firm system for generating and keeping intrapartum SB data as the largest data source comes from population-based household surveys (7, 8). Similarly, the quality of intrapartum care given to pregnant women at the health facility during childbirth in Ethiopia remains highly questionable and resulted in high figure (8).

Despite this, to prevent and curb this high rate, Every Newborn Action Plan (ENAP), a global plan aimed at improving neonatal health outcomes in 67 countries set to bring the rate to a level below 12/1,000 LBs by 2030 globally (27). Similarly, the Ethiopian government has been striving to improve maternal health care provision through increasing the number of facility deliveries, and training of health care providers (including midwives) at large in the past years. Apart from this, the Perinatal Death Surveillance and Response (PDSR) system was initially established in 2007 nationally and was later integrated into the Maternal Death Surveillance (MDSR) system and rechanged to the so-called comprehensive Maternal and Perinatal Death Surveillance and Response (MPDSR) system in August 2017 to better bring intrapartum SB into our eyes (28).

However, reducing intrapartum SB was found to be unachievable as planned in the past years. Growing evidence reveals that developing clear and successful interventions for the maternal and child health (MCH) program would be cost-effective to best prevent this high burden (8, 29). Many cross-sectional studies have been conducted on intrapartum SBs among women in Ethiopia, however, the results exhibit variations. To systematically analyze findings from various studies, we aimed to provide a nuanced understanding of the factors affecting intrapartum SB specific to the Ethiopian healthcare context. Investigating the factors influencing intrapartum SB will facilitate the development of better public health interventions to reduce these preventable deaths and improve maternal health. Therefore, the current systematic review and meta-analysis aimed to determine intrapartum SB and associated factors among women who had childbirth in Ethiopia using the available published evidence.

## Methods

### Study design and search strategy

The study used a systematic review of different published studies to determine the prevalence of intrapartum stillbirth and its determinants among women in Ethiopia. The registration number provided for this review is [CRD42024531247] in PROSPERO. To avoid possible duplications, we searched thoroughly for all studies published in Ethiopia on this topic. We reviewed all published studies in the following major databases: Medline/PubMed, Google Scholar, Scopus, Web of Science, Ethiopian University Repository Online, CINAHL, African Journals Online and Cochrane Library. The search for these published studies was not restricted by time, and all the published studies (articles) till Jan 1/2024 were considered and included. While identifying additional studies, genuine searching of the reference list of previous studies was also considered. We used some of the search terms such as “stillbirth OR intrapartum stillbirth among women OR determinants of stillbirth AND Ethiopia”. We followed the standard guideline of Preferred Reporting Items for Systematic Reviews and Meta-Analyses (PRISMA) when reviewing the studies.

### Study selection and eligibility criteria

This review mainly included studies that were conducted and published on intrapartum SB among women only in Ethiopia. All institutional (hospital) based studies were included. We also included studies that provided us with the prevalence of intrapartum SB and were published in English. Studies with home delivery were excluded from this review. All articles were assessed for inclusion firstly by looking at their title and abstract and if they met the criteria, a full review of papers was done to include this in the final review.

### Outcome of interest

The primary outcome of this review was the prevalence of intrapartum SB among women. The key determinant variables included in this review were residence (urban vs. rural), previous history of stillbirth, a high number of pregnancies, ANC follow-up, low birth weight, preterm birth, antepartum Hemorrhage, obstructed labor, preeclampsia, not using partograph and presence of obstetrical complication.

### Quality assessment and data collection

Joanna Briggs Institute Meta-Analysis of Statistics Assessment and Review Instrument (JBI- MAStARI) was used for the critical appraisal of the included studies. The overall quality of the articles for inclusion were assessed by two independent reviewers (AA, MW). Any misunderstanding or ambiguities between two independent reviewers on the overall quality of articles was resolved by adding a third independent reviewer and a thorough discussion. Questions to evaluate the methodological quality of studies on intrapartum stillbirth rate and associated factors in Ethiopia are the following:

Q1 = Was the sample frame appropriate to address the target population?
Q2. Were study participants sampled appropriately?
Q3. Was the sample size adequate as planned?
Q4. Were the study subjects and the setting described in detail?
Q5. Was the data analysis conducted with sufficient coverage of the identified sample?
Q6. Were the valid methods used for the identification of the condition?
Q7. Was the condition measured in a standard, reliable way for all participants?
Q8. Was there an appropriate statistical analysis?
Q9. Was the response rate adequate, and if not, was the low response rate? Managed appropriately?

We developed a data extraction tool that includes the name of the author/s, year of publication, study year, study- design, total sample size, study area, and the prevalence of stillbirth.

### Publication bias and heterogeneity

The publication bias and heterogeneity of the studies were assessed using Egger's and Begg's tests. The statistical significance of the publication bias was declared using a *p*-value < 0.05 while heterogeneity of the studies was assessed using *I*^2^ test statistics. 25%, 50%, and 75% of *I*^2^ test statistics were used to declare heterogeneity as low, moderate, and high respectively. The random effect model was also used as a method of analysis to reduce the heterogeneity of the studies for a given test result that indicates the presence of heterogeneity.

### Statistical methods and analysis

The data were first entered into a Microsoft Excel spreadsheet and then the extracted data was exported to STATA version 14 software for analysis. Random-effects model meta-analysis was used to calculate Ethiopia's intrapartum SB rate and associated factors. The subgroup analysis was also conducted by regions of the country and the effect of selected factor variables such as a previous history of intrapartum SB, ANC follow-up, low birth weight, preterm birth, antepartum hemorrhage, obstructed labor, preeclampsia, not using partograph, obstetric complications, the high number of pregnancy and residence on the intrapartum SBwas analyzed using separate categories of meta-analysis. The finding of the pooled prevalence was visually presented in a forest plot format with a *p*-value and a 95% confidence interval.

## Results

### Study selection

**Figure 1:**
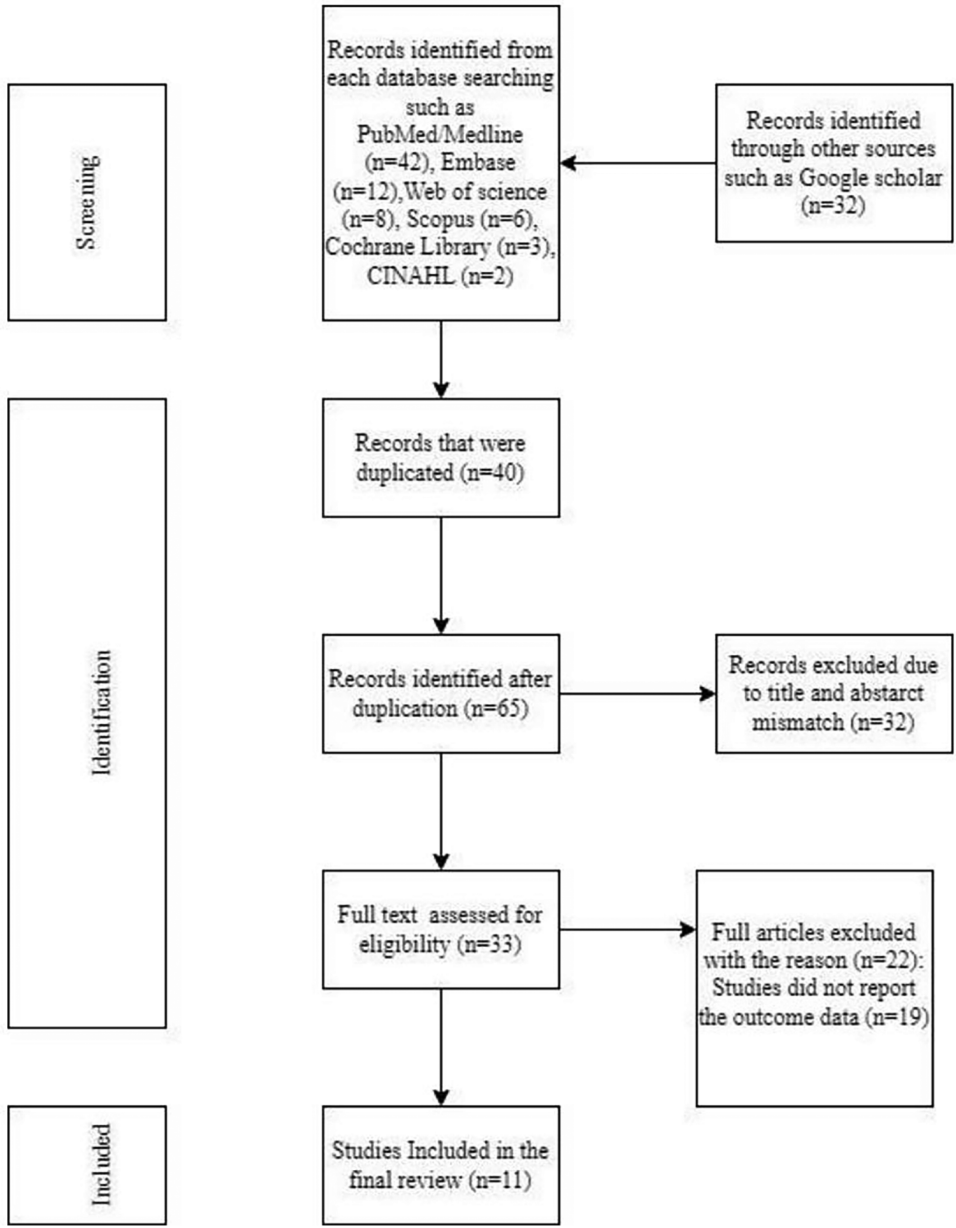
PRISMA flow diagram of the study selection process of studies on intrapartum stillbirth and associated factors among women who had childbirth in Ethiopia, 2024 (*n* = 11).

This review included published studies on intrapartum SB from various electronic online data searches of potential databases such as PUBMED/MEDLINE, Web of Science, Cochrane Library, Google Scholar, CINAHL, Scopus, Ethiopian University Repository Online, African Journals Online, and a manual search that yielded 256 records. The review took place from April to May 2024. Of those, 722 were duplicate records and were removed through screening using Endnotes and 148 were excluded after screening by title and abstract. A total of 36 full-text articles were screened for eligibility. From this, 25 articles were excluded since they conflicted with the inclusion criteria. Finally, eleven studies were included in the final quantitative meta- analysis (Figure 1).

### Quality appraisal of the included studies

The included articles were adequately assessed to know whether met the inclusion criteria or not. The quality of the articles was assessed before choosing the final review. A quality assessment indicator score of seven or higher was considered low risk for studies. The quality of the included studies assessed was more than 7, and there was low risk for all studies (Table 1).

**Table 1:** A table showing critical appraisal of included studies on the study of intrapartum stillbirth and associated factors among women who had childbirth in Ethiopia, 2024.

Name of the author	Q1	Q2	Q3	Q4	Q5	Q6	Q7	Q8	Q9	Total
Wolde et al. (15)	Y	Y	Y	Y	Y	Y	Y	Y	Y	9
Tilahun et al. (14)	Y	Y	Y	Y	Y	Y	Y	Y	Y	9
Mengistu et al. (18)	Y	Y	Y	Y	Y	Y	Y	Y	Y	9
Mulatu et al. (17)	Y	Y	Y	Y	Y	Y	Y	Y	Y	9
Berhe et al. (19)	Y	Y	Y	Y	Y	Y	Y	Y	Y	9
Mengesha and Dangiso (20)	Y	Y	Y	Y	Y	Y	Y	Y	Y	9
Ahmed et al. (16)	Y	Y	N	Y	N	Y	Y	Y	Y	7
Arega et al. (21)	Y	Y	N	Y	N	Y	Y	Y	Y	7
Beya et al. (22)	Y	Y	N	Y	N	Y	Y	Y	Y	7
Lolaso et al. (23)	Y	Y	Y	N	Y	Y	Y	Y	Y	8
Jamie (24)	Y	Y	N	Y	N	Y	Y	Y	Y	7

Whereas: Y, yes; N, no, Q1 = was the sample frame appropriate to address the target population? Q2-Were study participants sampled appropriately? Q3 -Was the sample size adequate? Q4—Were the study subjects and the setting described in detail? Q5 -Was the data analysis conducted with sufficient coverage of the identified sample? Q6 -Were the valid methods used for the identification of the condition? Q7-Was the condition measured in a standard, reliable way for all participants? Q8-Was there appropriate statistical analysis? Q9 -Was the response rate adequate, and if not, was the low response rate managed appropriately?

### Characteristics of included studies

This study included a cross-sectional study design that was conducted among women who had childbirth in Ethiopia. The minimum sample size reported in this study was 336 from a study conducted in Hiwot Fana Specialized Hospital, Ethiopia by Ahmed et al. in 2020 where whereas the maximum was 1980 from a study conducted in public hospitals in southwest Ethiopia by Lolaso T et al in 2021. The study included a total of 6,568 women. Almost all the studies were cross-sectional study design. More than one-third of the studies were from Harar regional studies, two were from Oromia and the other regions shared single articles (Table 2).

**Table 2:** Characteristics of studies included in the meta-analysis.

Name of the author	Year	Region	Study area/Health facility name	Study design	Sample size	Prevalence of intrapartum stillbirth% (95% CI)
Wolde et al. (15)	2019	South Ethiopia	Wolaita zone	Cross-sectional	737	9.0% (6.7%–10.8%)
Tilahun et al. (14)	2010	Oromia	Jimma University Hospital	Cross-sectional	413	8.0% (5.8%–10.2%)
Mengistu et al. (18)	2018	Harar	Hiwot Fana Specialized University Hospital	Cross sectional	557	14.5% (11.7%–17.6%)
Mulatu et al. (17)	2017	Harar	Hiwot Fana Specialized University Hospital	Cross-sectional	555	6.7% (4.7%–9.2%)
Berhe et al. (19)	2018	Tigray	Aksum General Hospital	Cross-sectional	570	3.6% (2.8–6.0%)
Mengesha and Dangiso (20)	2016	Sidama	Yirgalem General Hospital	Cross-sectional	370	9.2% (7.3%–12.4%)
Ahmed et al. (16)	2021	Harar	Hiwot Fana Specialized Hospital	Cross-sectional	336	12.5% (8.1%–14.6%)
Arega et al. (21)	2020	Amhara	Tibeb Gihon Specialized Hospital	Cross-sectional	366	3.8% (2.4%–5.3%)
Beya et al. (22)	2021	Oromia	North Shoa	Cross-sectional	348	13.9% (8.4%–16.8%)
Lolaso et al. (23)	2021	Southwest Ethiopia	Public hospital of south-west Ethiopia	Cross-sectional	1980	9.9% (8.5%–11.4%)
Jamie (24)	2022	Harar	University Hospital of Harar	Cross-sectional	336	12.6% (9.4%–14.2%)

### Prevalence of intrapartum Sb among women who had childbirth in Ethiopia

**Figure 2:**
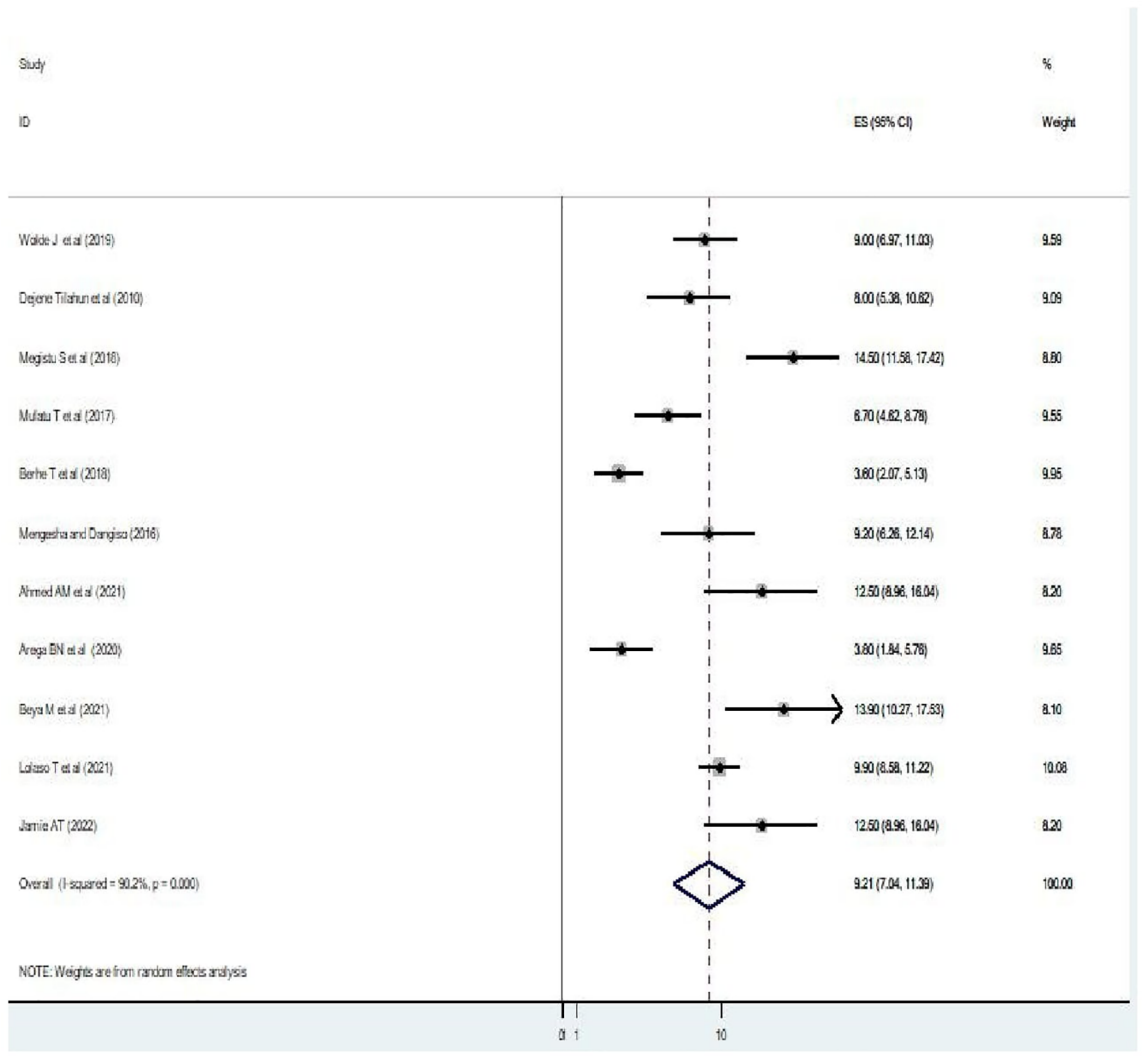
A figure showing a forest plot showing the pooled prevalence of intrapartum stillbirth among women who had childbirth in Ethiopia.

In the current review, the prevalence of intrapartum SB reported among women ranged from 3.6% to 14.5%. The pooled estimated prevalence of intrapartum SB among women who had childbirth in Ethiopia was 9.21%, 95% CI (7.03%–11.39%) with significant heterogeneity between studies (*I*^2^ = 90.2, *P* = 0.000) (Figure 2).

### Subgroup analysis of intrapartum Sb among women who gave birth in Ethiopia

**Figure 3:**
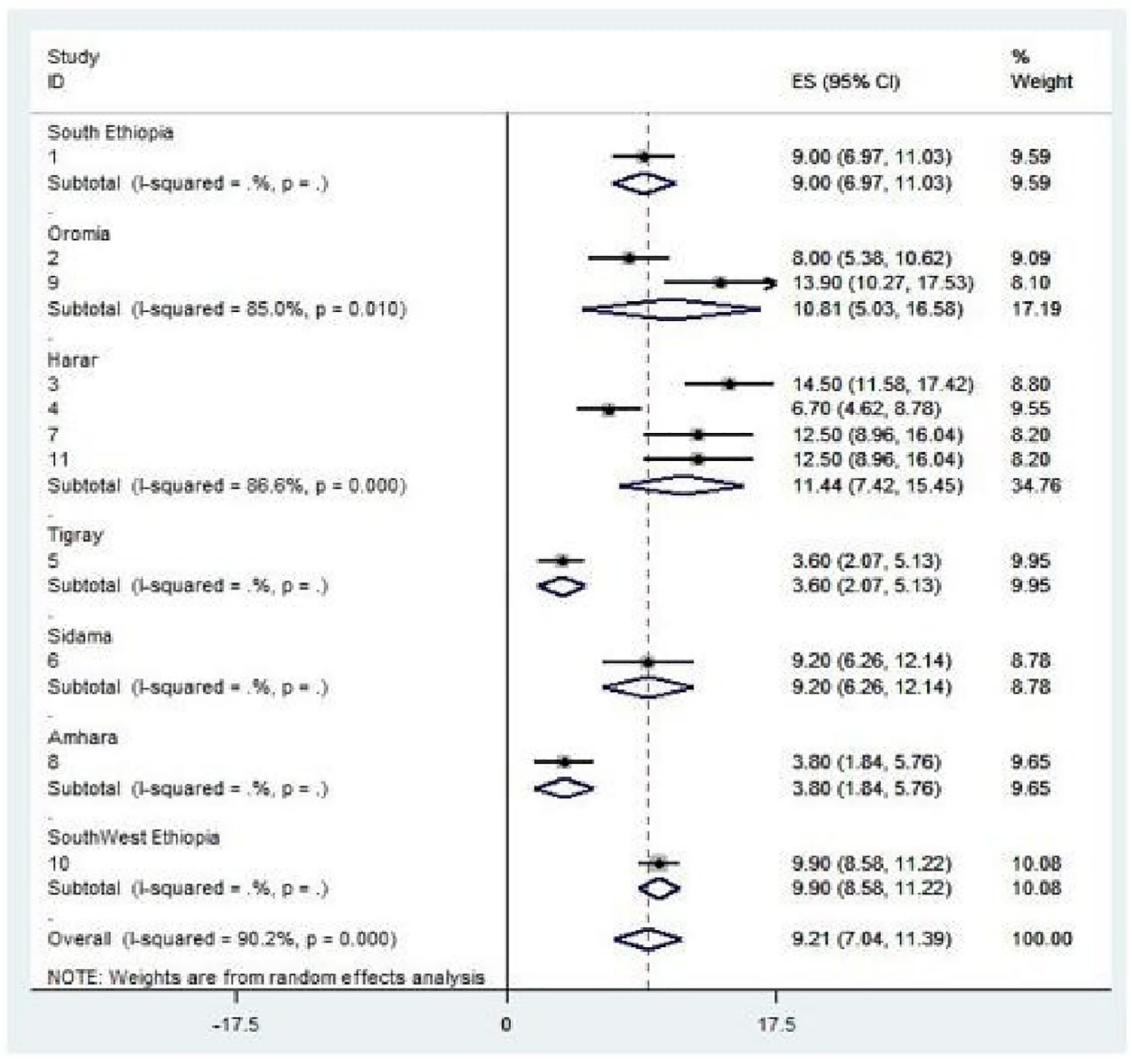
Subgroup analysis by regions on the intrapartum stillbirth and associated factors among women who gave birth in Ethiopia.

In this study, the subgroup analysis was performed in each region and the highest prevalence was observed in the Harar region with values of 11.44% (95% CI: 7.42, 15.45) and Oromia region with 10.81% (5.03; 16.58), followed by the Southwest Ethiopia region with 9.90% (8.58, 11.22). Contrary to this, the lowest values were observed in the Tigray region at 3.60% (2.07, 5.13) followed by the Amhara region at 3.80% (1.84, 5.76) (Figure 3).

### Heterogenicity and publication bias

**Figure 4:**
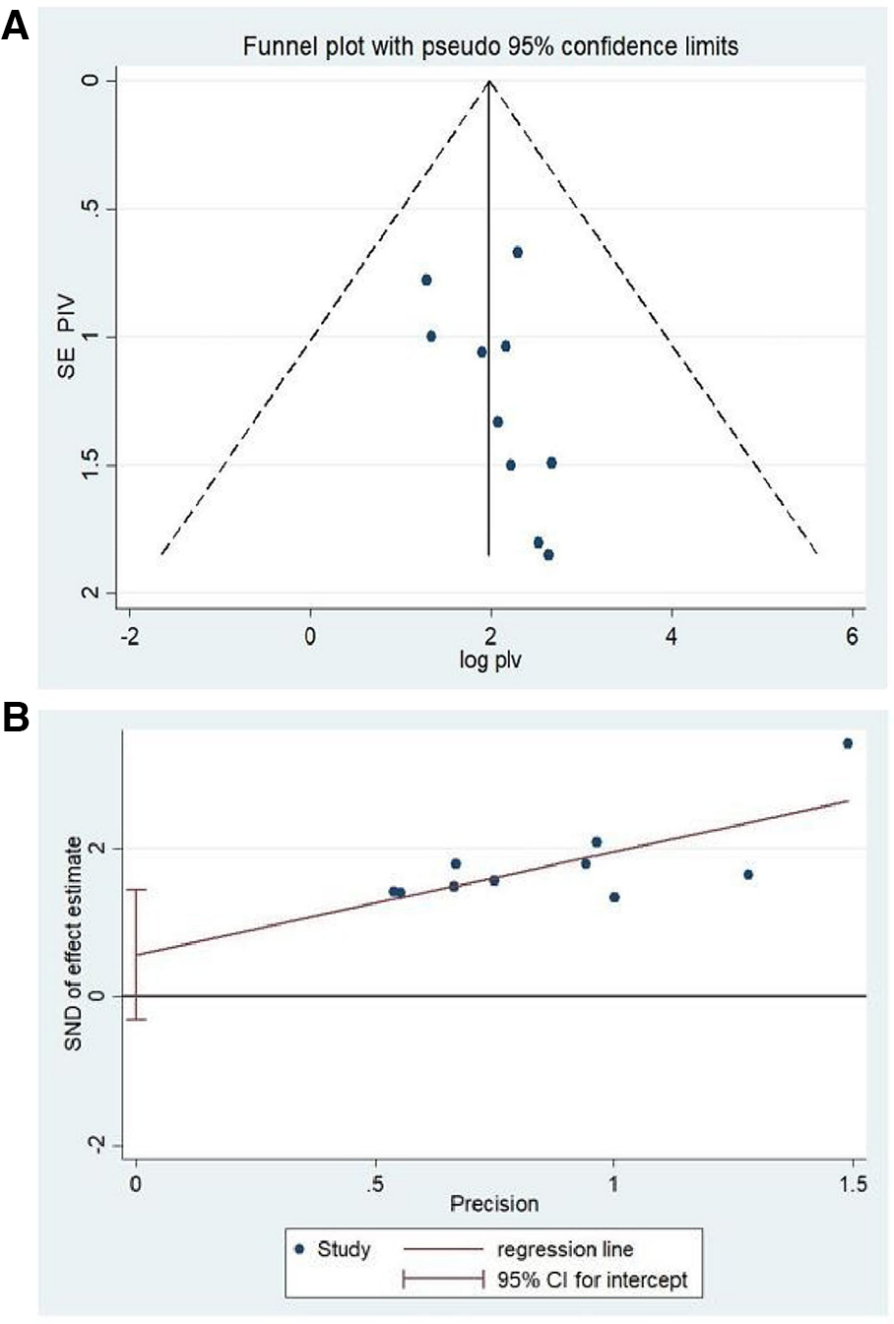
**(A)** A figure showing a funnel plot. **(B)** A figure showing Eggers test of the review.

To minimize and balance heterogeneity between studies, subgroup analyzes were done by region to examine whether the effect size varies across different subgroups. *I*^2^ test was primarily used to assess heterogeneity to check whether there is significant variation in prevalence between included studies and indicated that there was significant heterogeneity between reported studies (*I*^2^ = 90.2%, *P *≤ 0.000) (Figure 2). We then used random effect model to address the reported heterogeneity (variation between studies). The publication bias was examined through visual inspection of the funnel plot and Egger's test. Visual inspection with a funnel plot suggested that there is a symmetrical distribution among included studies (Figure 4A). Similarly, the result of Egger's test also showed that the test was not statistically significant for the presence of publication bias (*P* = 0.178) (Figure 4B). This indicated that both tests confirm that there was no publication bias for the current review. We used exclusion of studies method (excluding those studies that were deemed to yield high risk of bias to check for the individual effect on overall findings of the study) to assess for a sensitivity analysis and the results were found to be same (no significant change noted) and indicating the findings are so robust and reliable.

### Associated factors of intrapartum Sb among women who gave childbirth

**Figure 5:**
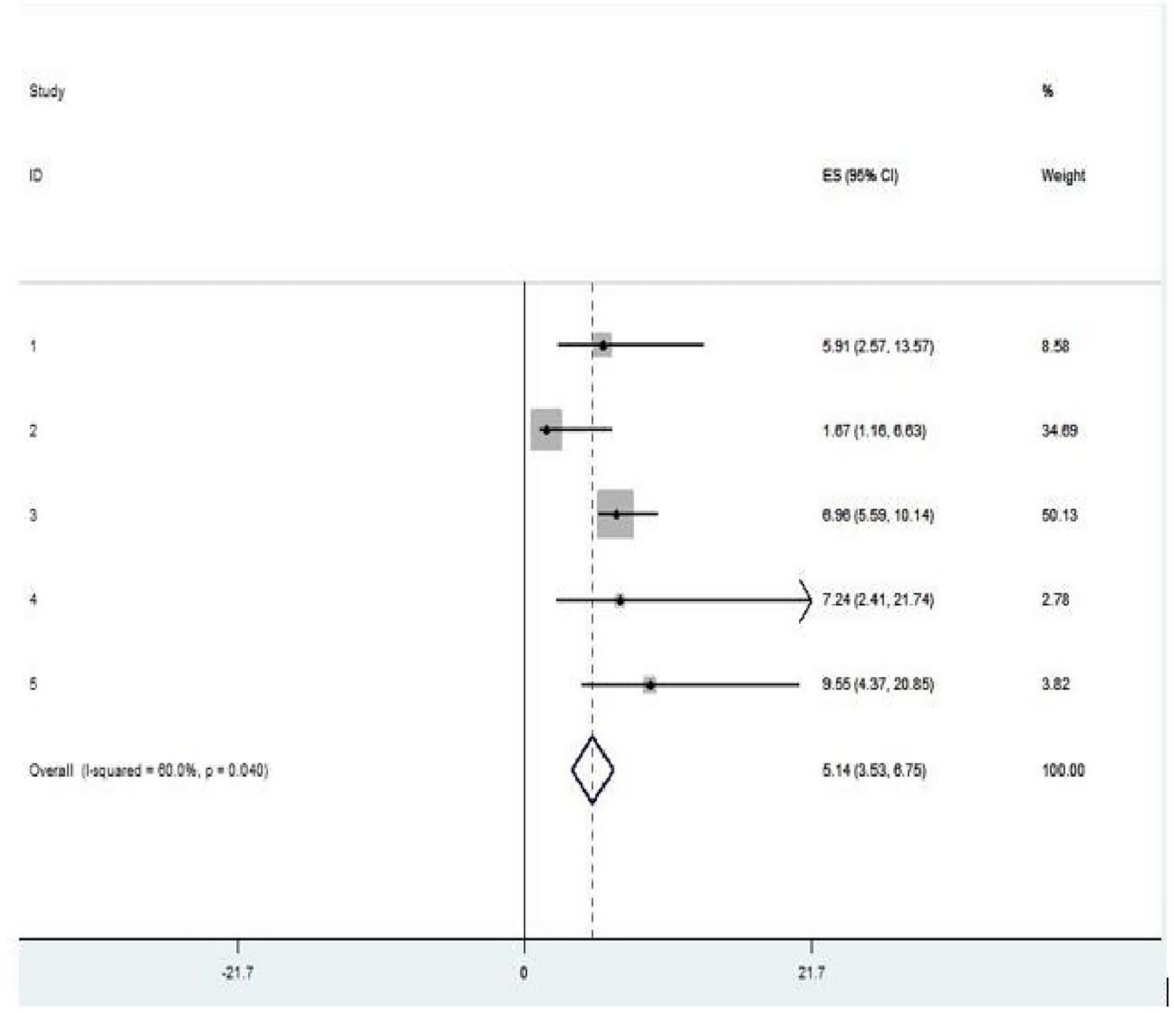
Pooled OR of the association between previous history of stillbirth and intrapartum stillbirth among women who had childbirth in Ethiopia, 2024.

**Figure 6:**
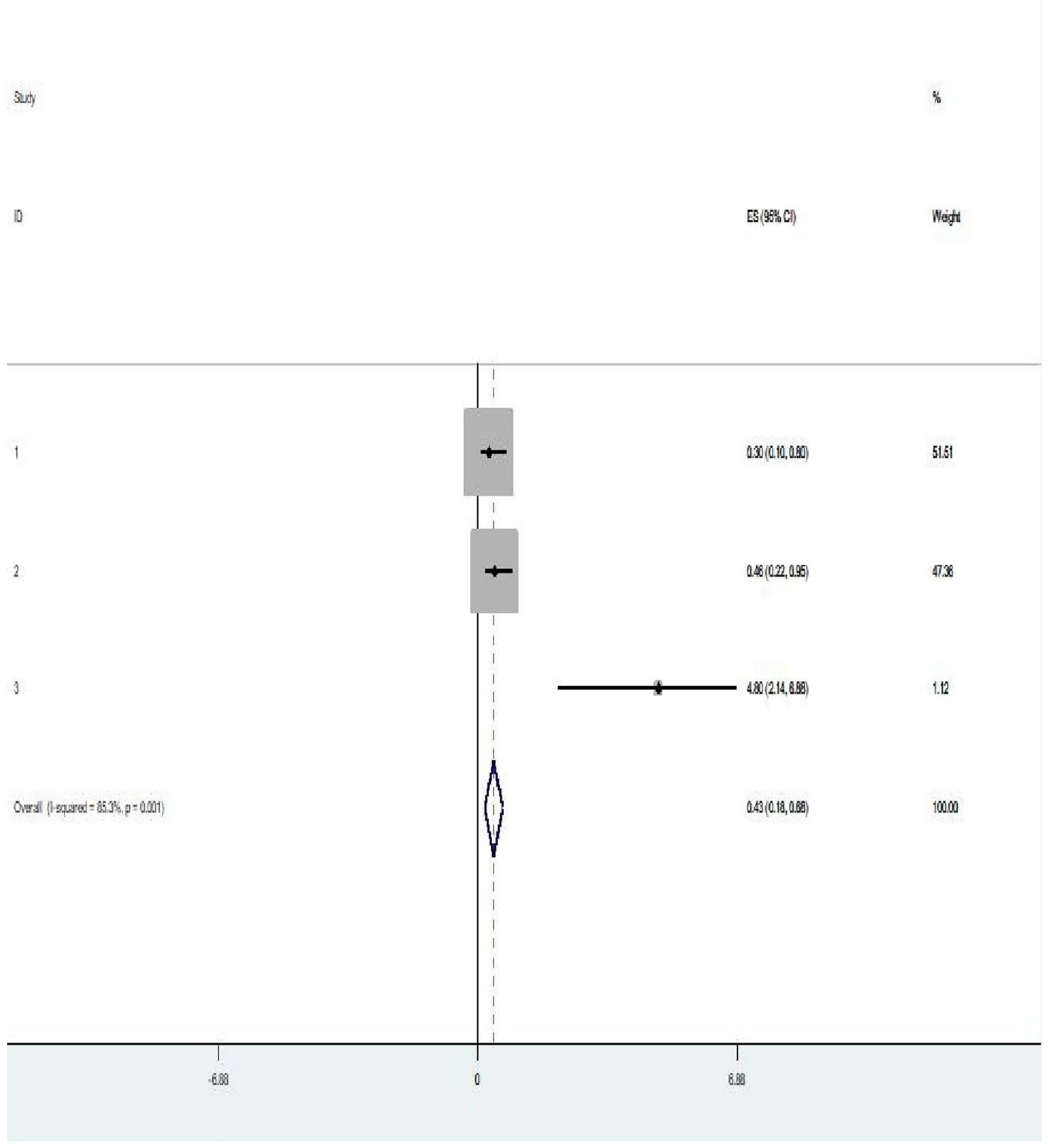
Pooled OR of the association between antenatal care use and intrapartum stillbirth among women who had childbirth in Ethiopia, 2024.

In the current review, two variables (previous history of intrapartum SB and no use of antenatal care) were significantly associated with intrapartum SB among women who gave childbirth (Figure 5). Women with previous history of intrapartum stillbirth had a fivefold increased probability of getting intrapartum SB when compared with their counterparts (OR = 5.14, *p *= 0.04, *I*^2^ = 60.0%) (Figure 5). This review also revealed that women who had not used antenatal care had 57% higher probability of developing intrapartum SB than those women who used antenatal care (OR = 0.43, *p* = 0.001, *I*^2^ = 85.3%) (Figure 6).

## Discussion

The current review was conducted to assess the pooled prevalence and determinants of intrapartum SB among women who had childbirth in Ethiopia using published studies. The pooled meta-analysis of the current review showed that the prevalence of intrapartum SB among women who gave birth in Ethiopia was quite high; 9.21%, 95% CI (7.03%–11.39%). This observed prevalence is higher than the studies conducted in Tanzania (3.5%) (30), Zambia (2.1%) (31), Ghana (2.39%) (29), Malawi (3.6%) (32), the United Kingdom (UK) (0.42%) (33),

West Africa (2.59%) (34), East Africa (0.86%) (35). This might be due to the observed variation in accessibility and utilization of maternal health care services, educational (literacy) level and other potential socioeconomic factors (9). In contrary to this, it is lower than systematic review and Meta analysis of SSA (21%) (36, 37). This could be due to a difference in sample size.

Women with a previous history of intrapartum SB had a fivefold increased probability of getting stillbirth when compared with their counterparts (OR = 5.14, *p *= 0.04, *I*^2^ = 60.0%). This could be due to the presence of a history of previous experience of adverse birth outcomes such as intrapartum SB putting the woman at greater risk of consequent pregnancy and that further results in intrapartum SB since poor obstetric histories are often recurrent and their causes are often unknown (38). This statement goes in line with a systematic review and Meta-Analysis conducted among high-income countries (3, 12). The current evidence in the field of preventive medicine suggests that pregnant women with a previous history of intrapartum SB should be counselled as early as possible for elective induction in the subsequent pregnancy at term preferably 37–38 weeks of gestation to decrease the risk of intrapartum SB occurrence (4, 34). The presence of structured and tailored interventions aimed at revitalizing the existing health extension package such as educating the woman about birth preparedness and complication readiness plan including how to detect potential risk, the importance of early ANC booking and encouraging the woman to have institutional delivery is still a cost-effective approach.

Having no antenatal care (ANC) attendance was also found to be associated with stillbirth in the current review. This goes with the studies conducted in Nepal and India (38, 39). This statement is supported by the evidence that the provision of good and quality ANC by skilled personnel with focused counselling on birth preparedness and complication readiness plans increases the utilization of health facility delivery and subsequently decreases the occurrence of intrapartum SB (40, 41). This statement is consistent with the study conducted in Ethiopia using the demographic health survey (DHS) data, which concluded experiencing intrapartum SB was significantly associated with the non-utilization of quality ANC (42, 43). Therefore, increasing access to pregnant women to have ANC would substantially decrease intrapartum SB that occurs due to non-utilization of ANC. This review also suggests strengthening the existing continuum of care approach (that starts from preconception care through the provision of quality antenatal and delivery service) and the use of community-based health extension workers (HEWs) to create awareness among women for early booking as well as training mid-level practitioners (midwives and other health professionals on basic and comprehensive obstetric care) and their heads for effective managerial skills would be cost-effective in this case (9, 13).

The current review used a more comprehensive search strategy, and more than one reviewer participated in each step of the review. PRISMA flow diagram was also strictly followed, and more than three-fourths of the region was represented in the current review. Despite this, it has some limitations. Firstly, all studies included in the review were cross-sectional and the outcome variable could be affected by other confounding variables. Secondly, some determinant variables were missed to fully show the factor for analysis. Thirdly, there was variability in sample size across studies. Fourthly, since these studies only included published data, publication bias might have been introduced. The current review calls for the need to assess the quality of ANC provision and tailor the need of target interventions to best improve the service quality.

Despite this, the current review tried its best to present the pooled estimate of stillbirth and its associated factors.

## Conclusions

Nearly one-tenth of women who had childbirth in Ethiopia had an intrapartum SB. Revitalizing the existing health extension package particularly family health services with much emphasis on focused antenatal care and counselling as well as with prompt referral system would reduce intrapartum SB. Regions with higher intrapartum SB prevalence should be given due emphasis and all stakeholders should also intervene in the identified factors to better reduce this high prevalence. This review calls for the need to assess the quality of ANC provision and tailor targeted interventions to best improve the service quality. Efforts should also be made for increased investment in maternal and child health services.
